# The impact of ChatGPT on human data collection: A case study involving typicality norming data

**DOI:** 10.3758/s13428-023-02235-w

**Published:** 2023-10-03

**Authors:** Tom Heyman, Geert Heyman

**Affiliations:** 1 Methodology and Statistics Unit, Institute of Psychology, Leiden University, Wassenaarseweg 52, 2333 AK Leiden, The Netherlands; 2 Nokia Bell Labs Antwerp, Copernicuslaan 50, 2018 Antwerpen, Belgium

**Keywords:** Large language models, ChatGPT, Human data collection, Typicality

## Abstract

Tools like ChatGPT, which allow people to unlock the potential of large language models (LLMs), have taken the world by storm. ChatGPT’s ability to produce written output of remarkable quality has inspired, or forced, academics to consider its consequences for both research and education. In particular, the question of what constitutes authorship, and how to evaluate (scientific) contributions has received a lot of attention. However, its impact on (online) human data collection has mostly flown under the radar. The current paper examines how ChatGPT can be (mis)used in the context of generating norming data. We found that ChatGPT is able to produce sensible output, resembling that of human participants, for a typicality rating task. Moreover, the test–retest reliability of ChatGPT’s ratings was similar to that of human participants tested 1 day apart. We discuss the relevance of these findings in the context of (online) human data collection, focusing both on opportunities (e.g., (risk-)free pilot data) and challenges (e.g., data fabrication).

The emergence of chatbots, and ChatGPT in particular, has had a profound impact on academia, both in terms of education and research (e.g., Kasneci et al., 2023; Pividori & Greene, 2023). Even though large language models (henceforth LLMs) like GPT and BERT have been around for some time (Devlin et al., 2018; Radford et al., 2018), recent advancements in combination with the development of a straightforward user interface (i.e., ChatGPT) presents academics with new challenges and opportunities. For example, its ability to generate or correct scholarly manuscripts raises questions about what constitutes authorship and how to distinguish between human and AI-based output.

This paper focuses on a different aspect that has received comparatively less attention, and that is the impact on (online) data collection (see also Dillion et al., 2023; Veselovsky et al., 2023). Given their remarkable performance on various tasks (see e.g., Bubeck et al., 2023; OpenAI, 2023), LLMs combined with an easily accessible user interface could be used to produce data that resemble those of human participants. On the one hand, this offers the possibility to study the architecture of LLMs and how they might relate to human psychology (Park et al., 2023). For example, Kosinski (2023) reported that output from LLMs resemble Theory of Mind abilities, though it is contested whether such findings should be considered as indicative of self-awareness (Marcus & Davis, 2023). On the other hand, if one is purely interested in collecting data from humans, the availability of tools like ChatGPT can be a reason for concern. Indeed, collecting data via online studies is becoming increasingly more popular in psychology (e.g., Sassenberg & Ditrich, 2019), but if one cannot distinguish AI-based output from human output, there is a risk of data contamination. In particular, longer tasks involving a repetitive action, such as in many norming studies for example, may be at risk (we revisit this issue in the discussion section).

The present study sought to examine this possibility by having ChatGPT perform a typicality rating task (e.g., *on a scale from 1 to 7, how typical is apple for the category fruit*). Previous research using LLMs to predict human typicality judgments for taxonomic categories, yielded mixed results. Seminal work by Connell and Ramscar (2001) found that similarities between exemplars (e.g., *apple*) and their respective category names (e.g., *fruit*) derived from latent semantic analysis (henceforth LSA) moderately correlated with typicality ratings. More recently developed, predictive language models have been shown to outperform count models like LSA on several tasks (Baroni et al., 2014; Mikolov et al., 2013), however Heyman and Heyman (2019) found that cosine similarity between exemplar embeddings and those of their respective categories were only weakly predictive of typicality norming data. Note though that results from Connell and Ramscar (2001) and Heyman and Heyman (2019) are not directly comparable, as they used different typicality data and the size of the categories was relatively small in the former study (as low as five exemplars for a given category). Furthermore, a recent study by Renner et al. (2023) managed to improve the prediction of the typicality ratings used in Heyman and Heyman (2019) considerably. Renner et al. (2023) used contextual language models, which entail that a word’s vector representation depends on the context, instead of remaining static as in Heyman and Heyman (2019). By applying *k*-means clustering to the contextual embeddings of exemplars and categories, Renner et al. (2023) were able to disambiguate the different possible meanings of words (e.g., *orange* can refer to a fruit or a color), thereby improving the typicality predictions.

Taken together, LLMs have been shown to predict human typicality judgments for taxonomic categories to a certain extent, but in all cases, some kind of processing was required to generate these typicality predictions. In contrast, the current study prompted ChatGPT to directly provide typicality ratings, which were then correlated with human norming data. Of course, this approach requires that ChatGPT is able to produce such output in the first place. So before undertaking the study described below, we verified whether ChatGPT is indeed capable of providing sensible, easily extractable ratings (i.e., within the specified range, say from 1 to 7), which indeed turned out to be the case. Furthermore, it is well documented that ChatGPT does not give the exact same answer to the same prompt (e.g., Reiss, 2023), which was also the case for typicality ratings.

We thus sought to explore whether ChatGPT’s output mimicked human typicality ratings. The aim was to answer three questions. First and foremost, to what extent (if any) are the typicality judgments generated by ChatGPT correlated with aggregated human typicality ratings? Secondly, how does ChatGPT’s item-total correlation compare to that of human participants? Put differently, if we would treat ChatGPT’s output as that of a regular participant and obtained aggregated typicality ratings across all participants (including those provided by ChatGPT), how would the correlation between ChatGPT’s ratings and the aggregate compare to the correlations between human participants’ ratings and the aggregate? Thirdly, we wondered how reliable ChatGPT’s ratings would be over time, or, in other words, what is the test–retest reliability of the ratings?

## Method

### Human typicality ratings

As in Heyman and Heyman (2019), we relied on English and Dutch typicality norms collected by Morrow and Duffy (2005) and De Deyne et al. (2008), respectively. Norms from the former only include summary statistics across participants (i.e., mean and standard deviation for every exemplar), hence we cannot answer the second research question as it requires access to every participants’ ratings. Luckily, De Deyne et al. (2008) did provide these data. More specifically, De Deyne et al. (2008) collected typicality ratings for exemplars of 16 taxonomic categories from second-year psychology students. Because the category *amphibians* only featured five exemplars, we did not consider it in the current study. The remaining 15 categories included between 20 and 33 exemplars (see Table 1), which were rated on a scale from 1 to 20 with 1 being *very atypical* and 20 being *very typical*. Every category’s exemplars were evaluated by 28 participants. Participants provided ratings for four categories, so in total there were 112 participants.

**Table 1:** Number of exemplars for each category

Category	Dutch	English
Animals		128
Birds	30	76
Clothing	29	92
Fish	23	
Flowers		52
Fruit	30	65
Furniture		42
Insects	26	48
Kitchen utensils	33	
Mammals	30	
Musical instruments	27	65
Professions	30	
Reptiles	20	
Sports	30	
Tools	30	64
Vegetables	30	31
Vehicles	30	72
Weapons	20	

Morrow and Duffy (2005) collected typicality ratings for exemplars of 11 taxonomic categories from healthy younger and older adults. The categories featured between 31 and 128 exemplars (see Table 1), which were rated on a seven-point typicality scale with 1 being labeled as *not at all*, 4 as *moderately*, and 7 as *extremely* typical. Every category’s exemplars were evaluated by 54 younger and 54 older adults, who provided ratings for all categories.

Note that for English, we used the norms of Morrow and Duffy (2005), rather than those recently collected by Banks and Connell (2022), because the former have already been used to evaluate LLMs (Heyman & Heyman, 2019; Renner et al., 2023), hence they allows us to better contextualize ChatGPT’s performance. Moreover, in Banks and Connell (2022), every exemplar was rated by a relatively small number of participants (i.e., 12), and each participant saw only a handful of exemplars from a given category. As such, these norms have limited utility when it comes to answering our research questions, which is no critique of their applicability in other contexts.

### ChatGPT’s typicality ratings

We entered the following prompt in ChatGPT to elicit typicality ratings mimicking the procedure of Morrow and Duffy (2005): “On a scale from 1 to 7 with 1 being not at all typical, 4 being moderately typical and 7 being extremely typical, how would you rate the typicality of the following concepts for the category [category label].” This was followed by a list of category exemplars in the order that they appeared in the norming data.

We used a similar approach, albeit with a slightly different prompt, to resemble the procedure and data from De Deyne et al. (2008): “On a scale from 1 to 20 with 1 being very atypical and 20 being very typical, how would you rate the typicality of the following concepts for the category [category label].” Note that instead of the original Dutch instructions and exemplar names, we used English translations as provided by De Deyne et al. (2008). The translations might not correspond to the same underlying mental representation (Malt et al., 2003), so it could introduce some noise and in turn deflate the correlation between the original judgments and ChatGPT’s output. On the flip side, the quality of the English output tends to be superior compared to other languages (Seghier, 2023), which could improve the correlation.

In some instances, two different Dutch exemplar labels had the same translation. Even though we entered those terms twice in the prompt, usually separated by several items, ChatGPT often removed the duplicate and provided only a single rating. In those situations, we decided to transfer the rating to the item with the same translation. On occasion, ChatGPT did not provide a rating for an exemplar. This sometimes happened for the last exemplar on the list, but overall the prevalence of missing data was quite low (i.e., *N* = 1 and 2, for the exemplars of De Deyne et al. (2008) and Morrow and Duffy (2005), respectively).

To generate typicality ratings, we used ChatGPT’s February 13 version with GPT 3.5. For both task instructions, we started a separate chat and asked to provide typicality judgments for every category in consecutive prompts, so not by editing an existing prompt and regenerating a response. In order to estimate the test–retest reliability, we repeated this procedure a few days apart, still using the same ChatGPT version, but again as two new chat sessions. On one occasion, so for one category, ChatGPT suddenly stopped generating output about halfway through the exemplar list. In this instance, we decided to regenerate the response. Other than that, we extracted the output as provided by ChatGPT and did not tweak the prompts to support a particular conclusion.

### Data processing and statistical analyses

ChatGPT’s responses were merged with the human typicality data. To answer the first research question, we correlated the mean of the human typicality ratings with ChatGPT’s ratings, for every category separately. We calculated both Pearson and Spearman correlations to facilitate comparison with previous work (Heyman & Heyman, 2019; Renner et al., 2023). All other correlations reported in the current paper are Pearson correlations. Note that for this analysis, we only used the initial responses by ChatGPT, so not the retest data.

**Table 2:** Correlations with mean human typicality ratings for each category

	Dutch			English Young			English Old	
Category	GPT_P_	GPT_S_	Best_H_	GPT_P_	GPT_S_	Best_R_	GPT_P_	GPT_S_
Animals				.61	.61	.70	.64	.64
Birds	.19	.27	.43	.42	.40	.53	.46	.36
Clothing	.48	.50	.39	.67	.69	.54	.61	.64
Fish	.56	.56	.50					
Flowers				.64	.55	.22	.52	.43
Fruit	.85	.86	.39	.61	.61	.47	.49	.49
Furniture				.79	.79	.76	.57	.65
Insects	.43	.33	.25	.66	.63	.41	.61	.56
Kitchen utensils	.61	.47	.17					
Mammals	.76	.78	.44					
Musical instruments	.92	.93	.49	.73	.76	.62	.76	.74
Professions	.38	.28	.07					
Reptiles	.54	.42	.20					
Sports	.76	.74	.29					
Tools	.31	.29	.46	.57	.52	.61	.66	.64
Vegetables	.23	.31	.31	.64	.66	.74	.56	.57
Vehicles	.79	.77	.73	.74	.71	.42	.68	.67
Weapons	.82	.86	.50					
*Mean*	.58	.56	.37	.64	.63	.55	.60	.58

*Note.* The columns labeled GPT refer to the correlation with ChatGPT’s output (subscript P for Pearson and S for Spearman). Best_H_ and Best_R_ show the correlations for the best performing predictor in Heyman and Heyman (2019) and Renner et al. (2023), respectively

The former were Pearson correlations, the latter Spearman correlations, as per the original papers

To answer the second research question, we first computed mean typicality ratings aggregated across human participants and ChatGPT’s output. Then, each individual set of responses, including ChatGPT’s, was correlated with those mean scores, again for each category separately. This yielded *N*+1 correlations per category; a correlation for all *N* participants, and one for ChatGPT’s ratings. In psychometrics, such correlations are often referred to as item–total correlations, though in the current context, we actually correlated the ratings of one *subject* with the aggregate of *all subjects* (including ChatGPT’s ratings). As was the case for the first analysis, we only used ChatGPT’s initial responses, so not the retest data. Recall that only De Deyne and colleagues’ (2008) norms made this kind of analysis possible, hence the results are based on their data from 28 human participants and one set of responses from ChatGPT. The main results of interests were ChatGPT’s item–total correlation, and how they compared to those of the 28 human participants. Note that for some categories, a few participants showed no variability in their response pattern, presumably because they were not paying attention. This was never the case for ChatGPT.

Finally, to answer the third research question, we calculated the correlation between the output provided by ChatGPT on two separate occasions, for each category (i.e., test–retest reliability).

**Fig. 1:**
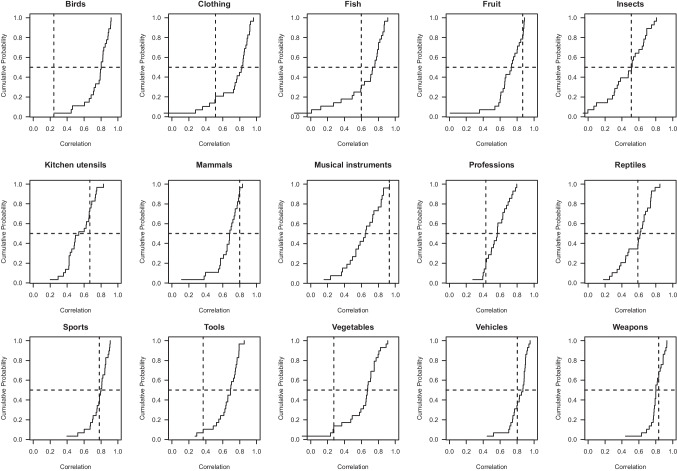
Distribution of item-total correlations per category. The value on the *y*-axis indicates the probability in the sample of observing an item-total correlation of less than or equal to the corresponding value on the *x*-axis. The *dashed vertical line* corresponds with ChatGPT’s item-total correlation for that category. The *dashed horizontal line* indicates the median. If these lines cross to the right of the step plot (e.g., for the category fruit), then ChatGPT’s ratings show a higher correlation with the aggregated typicality ratings than most human participants’ ratings do

## Results

### Relation with mean human typicality ratings

The correlation between ChatGPT’s output and the mean human typicality ratings are shown in Table 2 (columns labeled *GPT*). Results from previous studies (i.e., columns labeled *Best*) are included as a reference. On average, ChatGPT shows moderate correlations with the typicality norms of De Deyne et al. (2008) and Morrow and Duffy (2005). Compared to the results from Heyman and Heyman (2019), it offers a substantial improvement, and it even seems to do slightly better compared to Renner and colleagues’ findings (2023), though it does not have an edge in all categories. For example, the correlations are rather low for the category *birds*, and this seems consistent across norming data from two different languages. However, it should be pointed out that previous studies had some flexibility in selecting the best model/predictor for the typicality ratings, whereas the current study merely prompted ChatGPT to provide typicality ratings as if it were a regular participant.

### Item–total correlations

Figure 1 shows the magnitude of ChatGPT’s item–total correlation (i.e., vertical dashed line) and how it compares to that of human participants. For one category (i.e., *birds*), ChatGPT’s ratings yielded the lowest correlation with the average typicality ratings, whereas for another category (i.e., *musical instruments*), its correlation was higher than that of all 28 human participants. Across all categories, ChatGPT’s correlation was slightly below average, so in that sense its output is relatively similar to that of a regular participant in a typicality judgment task.

### Test–retest reliability

Table 3 shows the correlation between ChatGPT’s output on two separate occasions a few days apart. As can be seen, the test–retest reliability was quite high on average, yet never perfect. For some categories, the reliability was even fairly low, and this largely corresponded to worse correlations with average human typicality ratings (see Table 2), which is to be expected given Spearman’s correction for attenuation formula (Spearman, 1904). We cannot derive comparable outcomes from De Deyne et al. (2008) and Morrow and Duffy (2005) as they only collected data on one occasion. However, there are a number of other studies that did look at the test–retest reliability for similar taxonomic categories. More concretely, Barsalou (1987) found that test–retest reliability was about .92 when human participants were tested 1 h apart, .87 when the delay spanned a day, and .80 when there was 1–4 weeks in between testing sessions. As such, ChatGPT’s test–retest reliability estimates on average appear to be similar to those of human participants when surveyed 1 day apart.

## Discussion

The present study sought to establish to what extent ChatGPT’s typicality ratings for exemplars of taxonomic categories are similar to those provided by human participants. When it comes to the three research questions we had outlined, we can conclude that (1) ChatGPT’s ratings consistently correlated with aggregated human typicality judgments, (2) item–total correlations ranged from worse than all human participants to better than all human participants, with the average performance being similar to that of a typical participant, and (3) test–retest reliability was on average fairly high and similar to that of human participants tested on consecutive days.

Even though we did not directly ask ChatGPT to generate predictions of average human typicality ratings, it nevertheless mimicked norming data to a considerable degree, even outperforming previous predictions derived from LLMs for the same datasets (Heyman & Heyman, 2019; Renner et al., 2023). The purpose of the current paper is not to delve into the thorny issue of what underlies this (superior) performance, but rather to highlight the broader implications for conducting (online) studies involving human participants.

**Table 3:** Test–retest reliabilities of ChatGPT’s ratings for each category

Category	Dutch	English
Animals		.90
Birds	.69	.76
Clothing	.91	.85
Fish	.71	
Flowers		.77
Fruit	.94	.90
Furniture		.94
Insects	.79	.88
Kitchen utensils	.94	
Mammals	.84	
Musical instruments	.95	.92
Professions	.84	
Reptiles	.94	
Sports	.84	
Tools	.82	.81
Vegetables	.91	.70
Vehicles	.92	.85
Weapons	.93	
*Mean*	.86	.84

On the plus side, ChatGPT (or similar tools) could be used to generate or fine-tune instructions for studies. As such, it could be used in the piloting stage of a study before involving human participants, for instance, to check whether instructions or stimuli are unambiguous. Of course, there is a limit to what one can ask of ChatGPT, but it seems well equipped to handle text-based input such as typicality questionnaires. It is also conceivable that it will be able to process other modalities such as pictures in future versions (this is already the case for GPT-4, see OpenAI, 2023). Furthermore, one can even use the resulting data, such as those collected in the current study, to develop a (pre-registered) analysis protocol, which could in a later phase be applied to human data. A recent study even used GPT3.5 to generate data for 14 studies from the Many Labs 2 project, though for six of those it yielded (almost) no variability in its response pattern (Park et al., 2023).

On the flip side, LLMs and, ChatGPT in particular, can be misused by researchers to fabricate data. The same holds true for participants in (online) studies. The worry that online studies might yield suboptimal data is not new (see e.g., Webb & Tangney, 2022), but it is amplified with the introduction of tools like ChatGPT (e.g., Veselovsky et al., 2023). Consequently, researchers must take necessary precautions and design their studies in a way to encourage participants to answer truthfully, and discourage the use of ChatGPT or other means of generating unauthentic data (unless that would be the study’s goal). Norming studies that would require participants to provide a large number of judgments can be rather tedious. It is plausible that in such situations, some participant might be tempted to rely on ChatGPT, particularly if task instructions are easily extracted, and ChatGPT’s output can be entered in a straightforward fashion.

For example, performing a typicality rating task for a large number of categories by filling in an Excel file would be a prime example of a data collection procedure vulnerable to fabrication via ChatGPT. As the current study shows, quality checks that involve calculating the item–total correlation would not flag those results as problematic (except for the category *birds* perhaps). Similarly, ChatGPT did not respond in an identical fashion to the same query, so researchers cannot compare the ChatGPT output with the supposedly human-generated data. There are, however, tools to detect whether written content is likely created by a chatbot (Kirchner et al., 2023; ZeroGPT, 2023), but this mechanism is not applicable to output like ratings on a Likert scale, and the latter application is no longer available anyway due to its poor accuracy. So instead, researchers may need to consider including checks based on areas where ChatGPT is performing demonstrably worse than humans, yet there is no guarantee that this will remain the case. Perhaps more promising is to incorporate questions on which ChatGPT performs much better than humans, as long as it does not influence the task of interest, and does not take up too much time.

Better yet, when the aim is to collect human data, researchers should consider how to prevent the use of chatbots in the first place. For example, one could attempt to make the tasks more engaging, better explain the relevance of the study to participants, and offer fair compensation for their work (Cuskley & Sulik, 2022). These suggestions might not completely eliminate the possibility of data contamination, and they require careful consideration from the part of researchers. However, not adapting to the growing popularity of ChatGPT and similar tools, might be detrimental for online norming studies in psychological science and beyond.

### Additional considerations and limitations

The present study specifically focused on typicality ratings, but one might wonder about the generalizability of these findings to other tasks. For example, Loconte et al. (2023) evaluated ChatGPT on a number of tasks that reflect prefrontal functioning in humans, concluding that its performance ranged from average to impaired, relative to human participants. So, in the cases where ChatGPT systematically underperforms, it cannot be (mis)used to generate human-like data, or its application is at least limited in this regard. The same holds for situations where it outperforms human participants, and, to a lesser extent, when it shows no variability in response patterns when prompted repeatedly (as was sometimes the case in Park et al., 2023).

Furthermore, in tasks that involve a certain degree of subjectivity, such as the typicality rating task, there is no ground truth, and as such no obvious performance metric. Here, we used the degree of similarity to the aggregated human ratings as a proxy for performance. This only makes sense if there is a level of consistency in the data; in the current case, agreement about which exemplars are typical members of a category. Typicality ratings tend to be relatively consistent (Barsalou, 1987), yet people sometimes also disagree regarding the criteria for, say, a (typical) sport. Some might emphasize the physical element, whereas others focus on the rule-based aspect of activities (Verheyen & Storms, 2013). Such interindividual differences are not captured in the current analysis.

Besides generalizability across tasks, it is also uncertain how stable these findings will be over time. Indeed, we have seen remarkable improvements of the GPT model in the span of 6 years (OpenAI, 2023; Radford et al., 2018), so even though ChatGPT currently underperforms on certain tasks relative to humans, it is anyone’s guess what could happen in time.

Related to this notion of instability, it is important to point out that one cannot directly provide a seed to ChatGPT in order to generate reproducible results. However, it is possible to interact with the API, for instance through Python bindings, which, among other things, would allow one to specify the so-called temperature parameter. This parameter determines the extent to which responses are random, and setting it to 0 ought to make the responses mostly deterministic. However, given that the current study focuses on the implications of using ChatGPT in its default setting, we did not alter the temperature parameter. Furthermore, setting the temperature parameter to 0 would have prevented us from examining the test–retest reliability of the responses. As an aside, the default setting of the temperature parameter is 1 in the API, but it is uncertain which value is used for the ChatGPT interface.

Finally, it is possible that the norming data we used are part of the corpus on which the model underlying ChatGPT is trained. This contamination account has been used as a counterargument to claims that ChatGPT has mastered human capabilities like Theory of Mind (Marcus & Davis, 2023). Well-known experiments, including their rationale and normative responses, can be found on Wikipedia, which is typically part of the training corpus of LLMs. As such, it remains unclear whether the model has acquired particular skills or is merely paraphrasing training data. Interestingly, at some point ChatGPT, in addition to generating typicality ratings, did mention that these judgments are subjective and based on its training data. In doing so, it included a reference to Rosch and Mervis (1975). ChatGPT is known for generating bogus information at times (also referred to as hallucinating, see OpenAI, 2023), but in this case it might have referred to an actual paper. The paper in question is about graded structure, yet only describes data aggregated across category exemplars, which would not be directly informative for the current purposes. The paper does discuss typicality on a conceptual level; hence it is possible that ChatGPT somehow incorporated those principles.

Taken together, we cannot conclude that ChatGPT represents categories in a similar fashion as a typical human does. However, its output is not discernibly different compared to that of an average human participant. Regardless of whether this is the result of contamination, or a genuine development of human-like category structure, it presents researchers with opportunities (e.g., pilot-testing task instructions) and challenges. Most notably, it forces researchers to consider the possibility of data fabrication by participants in online studies, which might be hard to detect using current methods. Consequently, we encourage researchers to develop more sophisticated AI-detection mechanisms, and (re)design their studies in a way to elicit high-quality, authentic data.
